# Three decades of partnership: lessons from the Duke–open medical institute collaboration in global family medicine

**DOI:** 10.3389/fmed.2026.1866899

**Published:** 2026-07-29

**Authors:** John Anthony Vaughn, Stephanie Faschang, John Ragsdale

**Affiliations:** 1 Department of Family Medicine and Community Health, School of Medicine, Duke University, Durham, NC, United States; 2 Salzburg Stiftung der American Austrian Foundation Schloss Arenberg gem. Betriebs GmbH, Salzburg, Austria

**Keywords:** family medicine, global health partnerships, health profession education, health workforce development, primary health care (PHC)

## Abstract

Sustained international partnerships in health professions education are critical to strengthening global health systems that follow PHC principles, yet few models demonstrate long-term durability across changing institutional and geopolitical contexts. Drawing on semi-structured interviews with founding participants and archival program materials, this Perspective explores a three-decade partnership between Duke University’s Department of Family Medicine and Community Health (DFMCH) and the Open Medical Institute (OMI), examining how it emerged, how it has been sustained for 30 years, and what lessons it offers for global primary health care transformation through education. Established in the mid-1990s, the partnership evolved into a sustained educational exchange that introduced interactive learning models and fostered global networks among physicians from over 130 countries. The collaboration supports the development of global primary care capacity and pedagogical innovation in diverse settings while intentionally mitigating physician “brain drain” from developing countries. Key factors contributing to its longevity include shared mission, relational continuity, and an immersive residential learning environment. An iterative narrative analysis was used to identify key themes related to partnership formation, educational innovation, sustainability, and impact. This Perspective synthesizes these themes to offer insights into the design and durability of international collaborations in health professions education, as well as a transferable framework for developing sustainable global collaborations in health professions education.

## Introduction

Over the past three decades, global health has increasingly emphasized the importance of strengthening health systems that follow PHC principles (11, 13) through workforce development, educational innovation, and international collaboration (9). While many global health partnerships are time-limited, grant-driven, or narrowly focused, a growing literature has identified several characteristics associated with successful long-term collaborations, including shared mission, reciprocal learning, contextual adaptation, and sustained relationship-building (1–3). These studies demonstrate how educational partnerships can strengthen primary care capacity and faculty development across diverse settings. Nevertheless, relatively few published accounts examine collaborations that have endured across multiple decades and changing geopolitical contexts.

Several international primary care education initiatives provide useful examples. Talbot and colleagues described a Canada–Chile–Brazil partnership that used longitudinal capacity-building strategies to strengthen family health teams in Latin America (2). More recently, Wang and colleagues evaluated a Canada–China educational partnership designed to enhance leadership and teaching capacity among primary care educators (3). These initiatives demonstrated the value of educational exchange, faculty development, and local capacity building.

The collaboration between the Duke University Department of Family Medicine and Community Health (DFMCH) and the Open Medical Institute (OMI) builds on this tradition while offering a distinctive example of a sustained international partnership. Spanning more than three decades, the DFMCH–OMI relationship illustrates how educational exchange can evolve beyond knowledge transfer into a durable community of practice that supports professional development, cross-cultural learning, and primary health care transformation. What distinguishes the Duke–OMI collaboration is the durability with which these principles have been sustained across three decades of educational exchange.

Established in the mid-1990s in the aftermath of the Cold War, this partnership has brought together faculty and physicians from over 130 countries through immersive educational seminars in Salzburg, Austria. Designed to address disparities in access to medical knowledge and to strengthen health systems in developing countries, OMI created a platform for sustained, relationship-centered educational exchange. Participants were encouraged to stay connected with OMI, and, with continued commitment to their communities, were given opportunities to participate in ongoing seminars, externships, and scholarships. DFMCH was eager to partner and support these efforts.

This Perspective examines how the DFMCH–OMI collaboration builds upon themes previously identified in global primary care education partnerships to present a longitudinal case study in global health professions education and academic partnership. Drawing on oral history interviews with founding participants and archival program materials, we explore how this collaboration emerged, how it has been sustained over more than 30 years, and what lessons it offers for global primary health care transformation through education. In doing so, we argue that durable partnerships in global health must rest on a foundation of shared values, relational continuity, and pedagogical alignment.

## Approach

This article is informed by qualitative analysis of institutional memory and program documentation. Semi-structured oral history interviews were conducted with founding participants from both DFMCH and OMI leadership. These interviews explored the origins of the collaboration, key moments of challenge and adaptation, and reflections on long-term impact.

An iterative narrative analysis was used to identify key themes related to partnership formation, educational innovation, sustainability, and impact. This Perspective synthesizes these themes to offer insights into the design and durability of international collaborations in health professions education.

## Origins of a partnership in a transforming world

In the early 1990s, as the Iron Curtain fell and new democracies emerged across Central and Eastern Europe, a quiet but powerful form of diplomacy was taking shape through medicine. OMI, launched in 1993 by the American Austrian Foundation and the Open Society Foundations, sought to bridge the knowledge gap between East and West by inviting mid-career physicians to Salzburg for intensive week-long seminars with American and European faculty (7, 10). Among its earliest and most enduring partners was DFMCH, whose approach to holistic, community-based care would help define OMI’s philosophy for decades to come.

Duke’s introduction to OMI came through Dr. Mark Rogers, then Chief Executive Officer of Duke Hospital, who had completed a Fulbright fellowship in Austria and maintained ties with the American Austrian Foundation. He introduced OMI’s founder, Dr. Wolfgang Aulitzky, to DFMCH. At the time, academic family medicine in the United States was still consolidating its identity, and Duke’s department—led by Dr. George Parkerson (4) and soon after Dr. Lloyd Michener—was fighting for survival within the medical school.

The invitation to join OMI offered a chance to reaffirm the department’s purpose, demonstrate its value to the larger healthcare enterprise, and promote its community-oriented values internationally. For Aulitzky, Duke’s inclusion met an urgent need: “Family medicine in Europe is focusing on primary care collaborating closely with different medical specialists when needed. This is quite different from more comprehensive service offered by family medicine in the US system (8).” Duke’s first faculty delegation arrived in Salzburg in 1995, making it one of OMI’s earliest United States (US) partners.

With their shared missions, DFMCH and OMI proved a natural fit. OMI aimed to democratize medical education by bringing together physicians from 130 countries in shared residence at Schloss Arenberg. DFMCH’s model emphasized relationships, trust, and learning in community. “It wasn’t about exporting an American curriculum,” Michener (5) recalled. “It was about dialogue—learning from each other how primary care can serve real people.”

## Building and sustaining the partnership

A defining feature of the DFMCH–OMI collaboration has been its contribution to pedagogical innovation within the OMI seminar model. Duke faculty introduced small-group, interactive teaching approaches—including case-based discussions, role-play, and communication skills training—that contrasted with the predominantly didactic formats common in many participating countries at the time. Fellows are expected to participate in workshops in, among other topics, “difficult discussions in healthcare,” “narrative medicine,” and “goal setting.”

These formats not only enhance clinical skills in an innovative way, but also forge strong professional relationships throughout the week. These approaches not only align with principles of experiential learning, in which knowledge is constructed through active engagement and reflection, they also reflect the values of family medicine by emphasizing patient-centered communication, continuity of care, and attention to the social determinants of health.

The residential structure of the seminars further reinforced these educational principles. Participants and faculty live and learn together over the course of a week, creating an immersive environment that facilitates informal exchange, mentorship, and relationship-building. As one OMI leader described, “Everybody eats together, everybody teaches and learns together.” This structure fosters what Wenger (6) described as a *community of practice*, in which professional learning occurs through shared experience rather than hierarchical instruction. Faculty intentionally circulate to join different groups of fellows for meals throughout the week, and as relationships grow, conversations grow more engaged and livelier. Faculty recall evenings around the table when distinctions of title and country faded. “It was fun,” Michener said simply. “That’s why it lasted.”

## Sustaining collaboration over three decades

OMI is funded by a range of public and private institutions, corporations and foundations (10). The DFMCH–OMI collaboration has endured for decades despite the absence of substantial institutional funding or formal financial agreements from Duke. Several factors contributed to its durability.

First, the partnership was anchored in *shared values*, particularly a commitment to education as a relational and reciprocal process. Both institutions viewed teaching not as a unidirectional transfer of expertise, but as an opportunity for mutual learning and professional growth.

Second, *distributed ownership* within DFMCH allowed the collaboration to persist across leadership transitions. Responsibility for participation was maintained within the department rather than centralized at the institutional level, enabling continuity through faculty engagement and mentorship.

Third, the collaboration was sustained through *relational continuity*. Faculty and fellows developed enduring professional and personal connections through the immersive seminar experience. These relationships functioned as a form of social infrastructure, reinforcing commitment, and facilitating ongoing collaboration beyond formal program structures.

Finally, participants consistently described the experience as intrinsically meaningful and professionally renewing. As one faculty member noted, “It lasted because people believed in it—and because it was meaningful work.” This sense of purpose, combined with the enjoyment of teaching and learning in a global community, emerged as a critical factor in sustaining the partnership.

## Impact on health professions education and primary care

**Figure 1:**
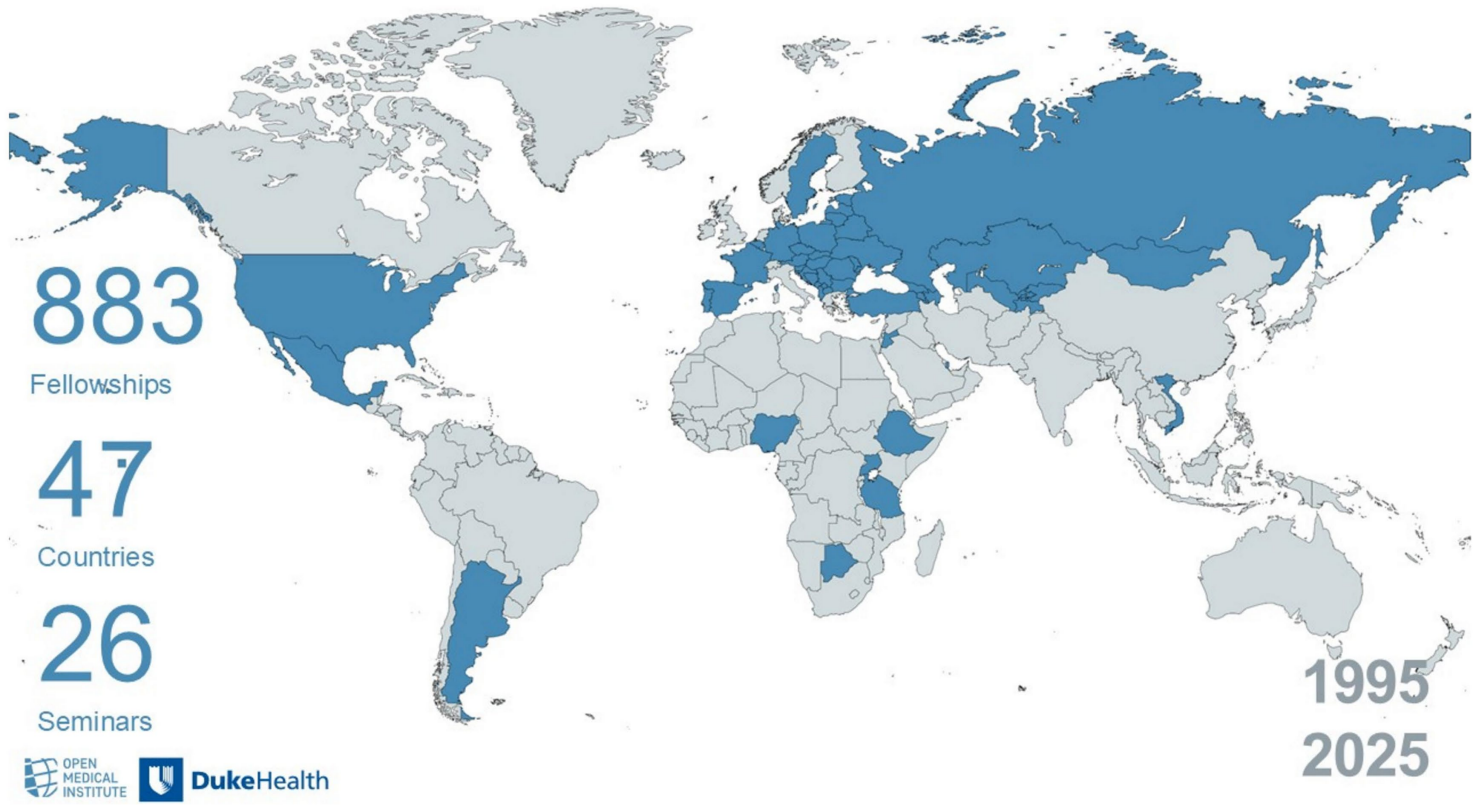
Global distribution of Duke-OMI family medicine fellows, 1995–2025.

Between 1995 and 2025, Duke Faculty led 26 family-medicine seminars at OMI, training 883 fellows from 47 countries. Evaluations and participant essays—nearly 900 documents—describe an exchange that continues to evolve as medicine globalizes. Fellows have returned to their home institutions equipped not only with updated clinical knowledge, but with new approaches to teaching, communication, and systems thinking. Importantly, the partnership challenges traditional assumptions about global health education as a one-way flow of knowledge from high-income to low and middle-income countries. Instead, it illustrates a model of reciprocal exchange in which all participants contribute to and benefit from shared learning Figure 1.

Reported impacts include the adoption of small-group teaching methods, incorporation of patient-centered communication practices, and development of primary care training programs in settings where such models were previously limited. Some participants have gone on to leadership roles in health systems and policy, extending the influence of the program beyond individual clinical practice.

For Duke Faculty, participation has functioned as a uniquely valuable form of ongoing faculty development. Teaching in cross-cultural environments requires clarity, adaptability, and humility, enhancing both pedagogical skill and cultural competence. The experience also fostered a global professional network that continues to influence teaching and collaboration.

Institutionally, the collaboration reinforces DFMCH’s commitment to addressing health in context—whether in Durham, North Carolina or Dubrovnik, Croatia. It demonstrates how an academic department can engage globally without major funding or bureaucracy, simply by showing up consistently with curiosity and respect. The OMI leadership sees Duke’s role as catalytic. “Family medicine gave OMI a conscience,” Aulitzky remarked. “It reminded everyone that medicine is about the patient’s life, not just the disease (8).”

## Lessons for global primary care health professions education

Three decades of the DFMCH–OMI collaboration offers several insights for the design of international partnerships aimed at strengthening primary health care education:

1. *Partnership as a learning system*: Effective collaborations function not as platforms for dissemination, but as environments for shared learning. Educational exchange is most impactful when participants engage as co-learners rather than as teachers and recipients.
2. *The importance of educational design*: Interactive, experiential learning approaches facilitate deeper and more durable transformation than traditional didactic methods. Educational design in which faculty and fellows actively participate is central to its impact.
3. *Relational infrastructure as a foundation for sustainability*: Long-term partnerships depend on trust, continuity, and shared experience. Structured opportunities for relationship-building over decades are essential components of successful collaboration.
4. *The role of family medicine in global health*: Family medicine’s emphasis on continuity, community context, and person-centered care provides a transferable framework for strengthening health systems that follow PHC principles across diverse settings (12, 13).
5. *Addressing physician “brain drain” through educational investment*: A foundational goal of OMI is to mitigate the migration of physicians from developing countries by strengthening professional capacity within local systems. The seminars aim to bring knowledge, skills, and networks to physicians in ways that can be applied in their home contexts. OMI’s intent was to “bring the knowledge to them, and in a way that has been informed by their preferences, so they do not have to leave to find it.” This approach reframes global health education as a strategy for retention and system strengthening.
6. *Modest resources, substantial impact*: Sustained educational partnerships do not necessarily require large-scale funding. Consistency, commitment, and alignment of values may be more important determinants of long-term success than financial investment alone.

These insights remain timely as academic centers face challenges with funding and so must reimagine global engagement after the pandemic era. As OMI digitizes its archives and DFMCH explores qualitative analyses of fellow outcomes, new opportunities arise to examine how educational exchange shapes health systems over time.

## Looking forward

As global primary care continues to grapple with workforce shortages, aging populations, and healthcare inequities, educational partnerships remain a central strategy for transformation. The DFMCH–OMI collaboration demonstrates that such partnerships can endure and evolve over decades when grounded in shared purpose, mutual respect, and a commitment to learning.

Beyond its historical significance, this collaboration offers a model for how academic institutions can engage globally in ways that are relational, reciprocal, and sustainable. In doing so, it underscores a fundamental insight: that the transformation of primary health care begins not only with systems and policies, but with the relationships among those who teach, learn, and practice within them.

Both partners are exploring ways to analyze 30 years of fellow evaluations and course materials using natural-language methods—a project that could quantify what participants have long described qualitatively: the transformation that occurs when clinicians learn not only new skills, but new ways of seeing.

For DFMCH, this history is a reminder that global health begins with relationships. As Michener reflected, “We learned that family medicine is not a discipline—it’s a way of being with people. That’s why it works anywhere.”

## Data Availability

Due to privacy considerations, no data can be shared beyond what is presented in the manuscript. Written informed consent for the publication of potentially identifiable data was obtained from participants.
